# Post-Contrast Acute Kidney Injury in Patients with Various Stages of Chronic Kidney Disease—Is Fear Justified?

**DOI:** 10.3390/toxins13060395

**Published:** 2021-06-01

**Authors:** Inga Chomicka, Marlena Kwiatkowska, Alicja Lesniak, Jolanta Malyszko

**Affiliations:** Department of Nephrology, Dialysis and Internal Medicine, Medical University of Warsaw, Banacha 1A, 02-097 Warsaw, Poland; inga.chomicka@wum.edu.pl (I.C.); mkwiatkowska1@wum.edu.pl (M.K.); alicja.lesniak@uckwum.pl (A.L.)

**Keywords:** acute kidney injury, iodinated contrast media, computed tomography, post-contrast acute kidney injury

## Abstract

Post-contrast acute kidney injury (PC-AKI) is one of the side effects of iodinated contrast media, including those used in computed tomography. Its incidence seems exaggerated, and thus we decided to try estimate that number and investigate its significance in our clinical practice. We analyzed all computed tomographies performed in our clinic in 2019, including data about the patient and the procedure. In each case, we recorded the parameters of kidney function (serum creatinine concentration and eGFR) in four time intervals: before the test, immediately after the test, 14–28 days after the test, and over 28 days after the test. Patients who did not have a follow-up after computed tomography were excluded. After reviewing 706 CT scans performed in 2019, we included 284 patients undergoing contrast-enhanced CT and 67 non-enhanced CT in the final analysis. On this basis, we created two comparable groups in terms of age, gender, the severity of chronic kidney disease, and the number of comorbidities. We found that AKI was more common in the non-enhanced CT population (25.4% vs. 17.9%). In terms of our experience, it seems that PC-AKI is not a great risk for patients, even those with chronic kidney disease. Consequently, the fear of using contrast agents is not justified.

## 1. Introduction

Intravenous iodine contrast agents have been used in medicine for over one hundred years, since 1923 when Osborne and colleagues from the Mayo Clinic performed urography [1]. The first reports of post-contrast acute kidney injury (PC-AKI) appeared in 1945, when Bartel and associates described anuria after pyelography [2]. Then, an avalanche of research began to identify risk factors, possible prophylactic measures, long-term effects of post-contrast acute kidney injury, and also attempts to reduce nephrotoxicity of iodinated contrast agents. It would seem that during these almost one hundred years it should have been possible to establish the principles of safe use of iodinated contrast media. The truth is that PC-AKI still remains a controversy that requires more research.

According to the latest ESUR Contrast Medium Safety Committee guidelines, post-contrast acute kidney injury is defined as an increase in serum creatinine concentration ≥0.3 mg/dL or ≥1.5–1.9 times the baseline value (AKI definition according to KDIGO) within 48–72 h after contrast medium administration. It is also worth noting that the guidelines distinguish the contrast-induced acute kidney injury (CI-AKI), which is similarly defined as PC-AKI, but diagnosis requires the exclusion of all possible causes of renal failure [3,4]. However, it seems that using this diagnosis in clinical practice carries a risk of bias, as we cannot absolutely exclude the influence of all potential nephrotoxic agents. It is also a major difficulty in determining the actual risk of kidney damage and the frequency of PC-AKI. 

In the first publications about PC-AKI, it was estimated that it occurs in up to 55% of people with reduced glomerular filtration. At that time, it was also claimed that the administration of iodinated contrast agents was the most common cause of AKI among hospitalized patients [5]. For this reason, very careful and strict qualification of patients for procedures requiring the administration of iodinated contrast media was performed, even for contrast-enhanced computed tomography. For example, a few years ago, the condition for the examination, even in patients with normal kidney function, was to withdraw metformin earlier, but now we know that it does not increase the risk of PC-AKI in this group and can be safely used [6,7]. It seems that these restrictions mostly affect patients with chronic kidney disease, which is an evident risk factor for the development of PC-AKI. There are no data on the number of cases in this group of patients where contrast-enhanced computed tomography was denied and diagnosis was delayed—we can only guess. A few years ago, a survey was conducted in which radiologists were asked about their clinical practice. According to that survey, up to 36% of radiologists declared that they do not perform contrast-enhanced CT in patients with multiple myeloma. Moreover, approximately 11% of the respondents answered that they did not perform contrast-enhanced CT in patients after kidney transplantation [8].

Currently, there are reports of a lower incidence of PC-AKI than before [5,9]. However, the unequivocal statement about the overestimation of the prevalence of PC-AKI requires confirmation in further studies. This gives hope for change in the current procedure and more liberal approach to qualification for contrast-enhanced tomography, faster diagnostics, and implementation of treatment. For this purpose, we first decided to estimate the incidence of AKI after computed tomography with iodine contrast in a small group of patients hospitalized in our department, as described in an earlier publication. We obtained interesting results that encouraged us to continue working in a larger group of patients [10].

## 2. Results

In 2019, a total of 706 computed tomographies were conducted at our Nephrology, Dialysis, and Internal Medicine Department. Of these, 128 examinations were performed on dialysis patients, including 92 contrast-enhanced CT and 36 non-enhanced CT. The above cases were not included in the further analysis, as assumed. After excluding examinations without subsequent control of renal parameters, we included 284 contrast-enhanced CT and 67 non-enhanced CT in the final analysis. At this stage, we can conclude that our department performed significantly more contrast-enhanced CT. It seemed that this was not characteristic of only 2019, but a similar disproportion was also present in previous years. However, we are not able to quote specific numbers as this was not the subject of our study. 

**Figure 1:**
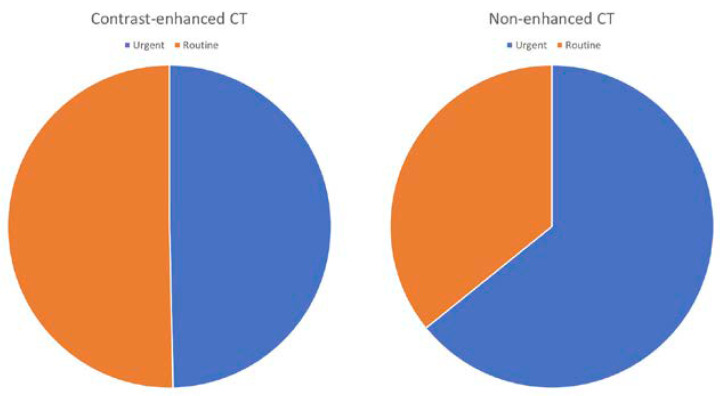
The figure shows the quantitative share of computed tomographies performed urgently (blue) and scheduled (orange). The graph on the right represents tomography performed with contrast enhancement (52.5% urgent), the one on the left without enhancement (35.8% urgent).

By analyzing the characteristics of the ordered computed tomography, we can conclude that in the case of contrast-enhanced CT, we performed urgent and routine examinations with similar frequency (143 vs. 141). However, non-enhanced CT was more often performed urgently than planned (43 vs. 24). (Figure 1) Efforts were made to maintain euvolemia and preserved diuresis before and after the examination in each patient undergoing contrast-enhanced CT. Taking into account the state of hydration and the patient’s burden, in some cases oral or intravenous hydration was used. In addition, potentially nephrotoxic drugs were discontinued 24 h prior to CT. In individual cases, acetylcysteine was also used. We also considered how many examinations were performed according to one protocol (i.e., only abdomen or chest or head etc.), two (i.e., abdomen + chest or chest + head or abdomen + head, etc.) or more. In the case of contrast-enhanced CT, 179 tests (63%) were based on one protocol, 86 tests (30.3%) on two, and 19 tests (6.7%) on more. Correspondingly, in CT without contrast enhancement, 58 tests (86.6%) were performed with one protocol, 7 tests (10.4%) with two, and 2 tests (3%) with more protocols. In our center, three types of iodine contrast agents were used: iomeprol, iopromide, and iodixanol. Two of them, iomeprol and iopromide, are low-osmolarity contrast agents, while iodixanol is the only iso-osmolar agent. After the analysis of the performed CT, we were able to safely assert that LOCM (low-osmolal contrast medium) was strongly preferred in our hospital—iomeprol was used in 149 examinations, and iopromide in 102 examinations. IOCM (iso-osmolal contrast medium) was only used in isolated cases—only seven iodixanol-enhanced examinations were performed in 2019. The average doses of iodine contrast agent were 76.99 mL for iomeprol, 78.43 for iopromide, and 88.57mL for iodixanol.

Moving on to the main assumption of our study, we looked at serum creatinine and eGFR in all patients who underwent computed tomography. We tracked the parameters of kidney function in all these patients at four time points: before computed tomography, 1–7 days after CT (mainly 1–2 days), 14–28 days after CT, and more than 28 days after the study. The mean serum creatinine level and eGFR were calculated for each time interval, as shown in the attached table (Table 1). We found 44 cases of AKI in patients undergoing contrast-enhanced CT, i.e., PC-AKI, representing 15.5% of all patients undergoing this type of CT. Similarly, in the case of non-contrast tomography, we found 17 cases of AKI, which is as much as 23.9%. The analysis of renal parameters among patients with AKI was performed analogous to the analysis in the entire study population. In this case, mean values of serum creatinine and eGFR were also calculated at the four time points mentioned above (Table 2). Both patient populations were of similar age—the mean age of patients after CT with contrast was 65.1, and for patients after CT without contrast, 71. The gender distribution was also relatively similar in both groups. Among patients undergoing contrast-enhanced CT, there were 135 women (47.5%) and 149 men (52.5%), while non-enhanced CT was performed on 35 women (52.2%) and 32 men (47.8%). We also analyzed the presence of comorbidities among patients, which in our opinion could have influenced the development of AKI, including PC-AKI. The table shows their incidence in both populations (Table 3). 

**Figure 2:**
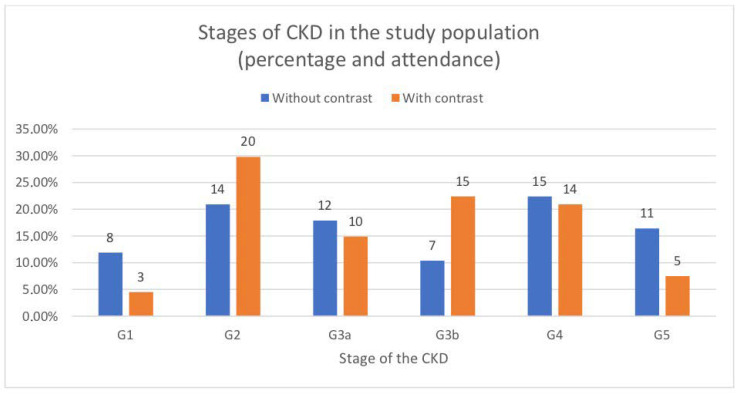
The figure shows the participation of chronic kidney disease stages in both study groups.

**Figure 3:**
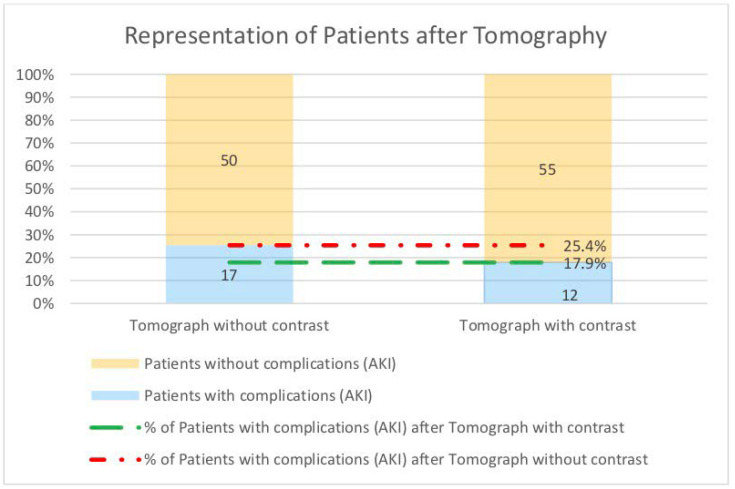
Incidence of acute kidney injury after non-enhanced and contrast-enhanced CT. The figure shows the percentage of the population diagnosed with AKI after the examination.

We realize that despite the above-mentioned similarities, due to the disproportion in numbers, we cannot draw a firm conclusion about the higher incidence of AKI after non-enhanced CT. Due to this fact, we tried to choose patients of similar sex, age, and number of comorbidities in both groups (contrast-enhanced CT and non-enhanced CT). On this basis, two groups with similar characteristics were created, representing different stages of CKD defined according to KDIGO [11] (Figure 2). The incidence of AKI after CT was reanalyzed, resulting in 12 cases of AKI after contrast-enhanced CT (which is 17.9%) and 17 cases of AKI after non-enhanced CT (which is 25.4%) (Figure 3). This is quite surprising as it suggests that contrast administration and its effect on the kidneys does not significantly increase the incidence of AKI in hospitalized patients. We analyzed the indications for CT in both study groups, mostly finding life indications, which confirms our assumption that patients were referred for examination on the basis of indications and not on parameters of kidney function (Table 4 and Table 5).

Regardless of whether or not iodine-based contrast agents were administered, we can clearly state that greater risk of AKI occurs after performing emergency tomography. Among the CT with administration of iodinated contrast media, there were 10 cases of AKI out of 37 emergency examinations (estimated risk of AKI 27%) and 2 cases of AKI out of 30 routine examinations (estimated risk of AKI 6.7%). A similar situation occurred in the case of emergency non-enhanced CT, where 12 cases of AKI were registered out of 43 tests performed (estimated risk of AKI 27.9%). Noteworthy is the number of AKI cases after routine CT without contrast administration—as many as 5 cases of AKI were registered out of 24 examinations (estimated risk of AKI 20.8%) (Table 6). The type of contrast used probably did not influence the development of AKI. In the contrast-enhanced CT that were included in the final analysis, two types of iodinated contrast media were used: iopromide and iomeprol. AKI was found in approximately 19.2% of the CT with administration of iopromide and 18.2% with administration of iomeprol.

**Figure 4:**
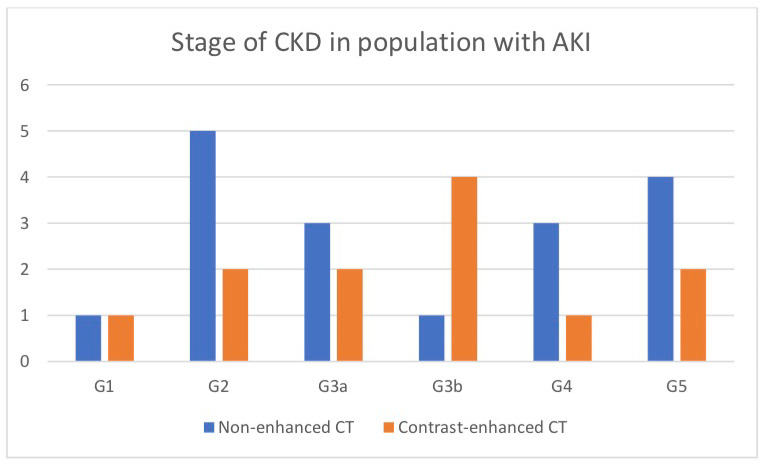
Number of patients at particular stages of chronic kidney disease who were diagnosed with AKI after computed tomography (including whether iodinated contrast media was administered).

Additionally, we assessed patients in both study groups who developed acute kidney injury in both study groups (contrast-enhanced CT and non-enhanced CT), in terms of age, number of comorbidities, and the stage of chronic kidney disease. Thus, we can conclude that one of the factors increasing the risk of AKI after CT is advanced age (above the age of 70, according to our study). In addition, it seems that the number of comorbidities did not increase that risk. On average, in the group of patients undergoing non-enhanced CT, we found four comorbidities, and in the group of patients undergoing contrast-enhanced CT, we found 3.9. For both groups that developed AKI, the mean number of comorbidities was 4.4, regardless of contrast was administration. After analyzing the number of AKI episodes in specific stages of chronic kidney disease (CKD), we found no obvious relationship between the two. An interesting finding is the fact that 58.3% of people with AKI after contrast-enhanced CT had advanced CKD before the procedure (i.e., G3b, G4, and G5 stages). On the other hand, in patients undergoing non-contrast CT, the percentage of cases with advanced CKD that developed AKI was 47.1%. This was not a large difference, and thus in order to unambiguously associate the higher incidence of AKI in advanced CKD after contrast administration, we argue that the study group should be larger (Figure 4). The group in the G3b stage seemed to be most interesting because only in this group did we find more frequent AKI cases after contrast-enhanced CT. However, justifying this thesis would require research on a larger population.

## 3. Discussion

In our study, we assessed patients in both study groups who developed acute kidney injury in both study groups (contrast-enhanced CT and non-enhanced CT), in terms of age, number of comorbidities, and the stage of CKD per KDIGO definition [11]. Thus, we can conclude that one of the factors increasing the risk of AKI after CT is advanced age (above the age of 70, according to our study). In addition, it seems that the number of comorbidities did not increase that risk. On average, in the group of patients undergoing non-enhanced CT, we found four comorbidities, and in the group of patients undergoing contrast-enhanced CT, we found 3.9. For both groups that developed AKI, the mean number of comorbidities was 4.4, regardless of contrast, was administration. After analyzing the number of AKI episodes in specific stages of CKD, we found no obvious relationship between the two. An interesting finding is the fact that 58.3% of people with AKI after contrast-enhanced CT had advanced CKD before the procedure (i.e., G3b, G4, and G5 stages). On the other hand, in patients undergoing non-contrast CT, the percentage of cases with advanced CKD that developed AKI was 47.1%. This was not a big difference, and thus to unambiguously associate the higher incidence of AKI in advanced CKD after contrast administration, we argue that the study group should be larger (Figure 4). The group in the G3b stage seemed to be most interesting because we found more frequent AKI cases after contrast-enhanced CT only in this group. However, justifying this thesis would require research on a larger population. 

In addition, we would like to stress that the rise in serum creatinine seen at more than 28 days in the contrast group could not be attributed to the contrast administration. Data were collected form the medical charts from the hospital or outpatient charts (another hospitalization, infection, dehydration, worsening of heart failure, acute coronary syndrome, etc.). Therefore, they were not relevant to the CI-AKI. 

Our study indirectly demonstrated that the problem of PC-AKI after computed tomography seems to be exaggerated. This finding is also supported by two large meta-analyses, where a total of 19,000 patients were examined, and the incidence of PC-AKI was estimated to be 5.0–6.4%, which is significantly lower than previously thought [12,13]. In these two papers, first of all, serum creatinine was measured in the vast majority after 2–3 days [12,13,14], while we did measure creatinine immediately after the CT, i.e., within 1–2 days, and thus several cases could have been missed in the studies included in the meta-analysis [12]. In addition, some of the studies were small, with 19 or 20 patients. In the largest study included in the meta-analysis on 11,516 patients with serum creatinine assessed after 7 days, CI-AKI incidence was 11.7% [13]. In the recent KOMPAS trial assessing the renal safety of omitting prophylactic prehydration prior to iodine-based contrast media administration in patients with stage 3 CKD, PC-AKI occurred in 11 patients (2.1%), including 7 of 262 (2.7%) in the no prehydration group and 4 of 261 (1.5%) in the prehydration group [15]. They evaluated serum creatinine after 2 to 5 days after contrast administration, and therefore their incidence of PC-AKI was relatively low as they may have missed several cases [15]. In our study, we assessed the incidence of acute kidney injury in relation to contrast administration. In the NICIR study, PC-AKI rate was 4.4% (95%CI: 1.4–9.9%) in the oral hydration arm and 5.3% (95%CI: 2.0–11.1%) in the i.v. hydration arm [16]. They also assessed serum creatinine within 48–72 h after the procedure. In the study by Chaudhury et al. [17] from the Cleveland Clinic CKD registry, the incidence of AKI was 27% in the coronary angiography group, 24% in CT with contrast, and 24% in CT without contrast. The incidence of AKI in CT without contrast was similar to our study and higher than in CT with contrast. Hinson et al. [18], using the Acute Kidney Injury Network/Kidney Disease Improving Global Outcomes criteria, found that the probabilities of developing acute kidney injury were 6.8%, 8.9%, and 8.1%, in the contrast-enhanced CT, unenhanced CT, and non-CT groups, respectively. They measured serum creatinine 48–72 h after the procedure. In another study [19] in septic patients, the incidences of AKI were 7.2%, 9.4%, and 9.7% in those who underwent CECT, unenhanced CT, and no CT, respectively. In both studies, the authors measured serum creatinine 48–72 h after the procedure. CM administration was not associated with an increased incidence of AKI. The authors concluded that their findings argued against withholding CM for fear of precipitating AKI in potentially septic patients. In a recent study, Gorelik et al. [20] reported that rate of AKI in patients undergoing CT with contrast with eGFR < 30 mL/min/1.73 m^2^ was 36%, while in eGFR > 30 mL/min/1.73 m^2^ was 7.6%, and in eGFR 30–44 mL/min/1.73 m^2^ was 19.8%. The rate of AKI in CT without contrast in patients eGFR < 30 mL/min/1.73 m^2^ was 24.4%. In their previous retrospective assessment of renal outcome in 12,580 hospitalized patients undergoing contrast-enhanced CT, the rate of AKI was 8% [21]. In the prospective study of 1009 participants in the Swedish CArdioPulmonary bioImage Study (SCAPIS), PC-AKI was observed in only 1.2% according to the old ESUR criteria (>25% or >44 μmol/L Scr increase) with creatinine measurement in 2–4 after the procedure [22]. Fukushima et al. [23] reported the incidence of CIN of 5.1% in 267 patients with eGFR below 60 mL/min/1.73 m^2^ within 3 days post-CT with contrast. Ellis et al. [24] reported that in patients with an eGFR less than 30 mL/min/1.73 m^2^, the proportion with post-CT AKI was 35% in contrast-enhanced CT and 14% unenhanced CT. However, in patients with an eGFR of 30–44 mL/min/1.73 m^2^, the proportion with post-CT AKI was 16% in the patients undergoing contrast-enhanced CT and 15% in the patients undergoing unenhanced CT. The higher rates of AKI than in our study (31% in the contrast vs. 34% in the non-contrast group) were reported recently in ICU patients by McDonald et al. [25]. Relevant studies are summarized in Table 7.

In addition, recently, there have been several studies in which the incidence of AKI was similar when compared with patients after contrast-enhanced computed tomography and non-enhanced computed tomography. As in our study, there was no evidence that the administration of an iodinated contrast media had an obvious effect on kidney function [26,27]. We look forward to the results of the INCARO study, the first randomized study on the effect of iodinated contrast agents in computed tomography on kidney function [28]. Due to the randomization and prospective design of the study, there is a chance that it will be the first referential assessment of the incidence of PC-AKI. However, until the end of the study (the recruitment of patients is estimated at 3 years), we must rely on observational and retrospective studies such as ours.

At this point, we should consider the reason for the dramatic difference in the approach to post-contrast kidney injury. The first explanation may be the change in the osmolarity of the contrast agents. Initially, high-osmolar contrast media (HOCM) with proven nephrotoxic effects were predominantly used. The mechanism of PC-AKI is twofold: there is direct damage to the cell membrane of epithelial and endothelial cells and vasoconstriction through the release of cytokines and secondary hypoxia. Currently, iso-osmolar (IOCM) and low-osmolar contrast media (LOCM) are preferred, and thus such severe side effects are not observed [5,9,27]. Comparing the above-mentioned types of contrast agents, we found no significant differences in their influence on the development of PC-AKI. In addition, when analyzing the occurrence of PC-AKI, we should pay attention to two other risk factors connected to the procedure itself, i.e., the amount and method of administration of the contrast agent. The administration of contrast agents intravenously, as in computed tomography, is much safer than intra-arterial administration, which takes place during endovascular interventions. Interestingly, it seems that the amount of contrast media administered probably does not play a significant role in intravenous administration [3,29].

Our goal is not to disregard the nephrotoxic effect of iodine contrast agents; rather, we would like to emphasize the need to change and standardize the current procedures, especially when performing computed tomography. At present, the ESUR Contrast Medium Safety Committee guidelines recommend the use of the eight-variable Mehran score to assess the safety of iodinated contrast media and prophylactic intravenous rehydration in people at risk of PC-AKI, such as renal failure (defined as eGFR < 60 mL/min/1.73 m^2^) [7]. Interestingly, although many studies have shown the nephroprotective effect of prophylactic hydration, the last large, randomized trial AMACING not only did not demonstrate the effectiveness of this procedure, but instead in some cases even proved its harmfulness [30]. 

### 3.1. Study Limitations

First, as serum creatinine was assessed in patients undergoing CT with or without intravenous contrast agent administration, we cannot extrapolate our results to coronary angiography or to percutaneous coronary interventions with intraarterial contrast administration. In addition, we studied changes in serum creatinine within 1–7 days, mainly 1 or 2 days, after CT to capture the early rise in serum creatinine as per definition. Many studies assess serum creatinine within several days. Second, our study was retrospective and single-center, but real-world data were gathered. In randomized controlled trials, patients represent selected populations with better compliance. In patients undergoing CT, possible nephrotoxic drugs, i.e., (NSAIDs, diuretics, biguanidine derivatives in diabetic patients) were withdrawn, and RAAS blockers were either withdrawn (when blood pressure permitted) or halved 24 h before the procedure (in elective CT), whereas aminoglycosides were administered extremely sparingly in our department. Among euvolemic and hypovolemic patients, whose clinical course permits it, we administer intravenous isotonic saline between 0.5 and 1 L before and after the procedure (time permitting), in total 1–2 L. We do not use acetylcysteine as a preventive measure. Hypervolemic patients and patients receiving dialysis in general are not given volume expansion (dialyzed patients were excluded from the analysis). The real-world data presented in our study could be either limitation or strength. Our findings may have important implications for the clinical management of patients undergoing CT. The “window of opportunity” is narrow in contrast nephropathy, and time is limited to introducing proper treatment after initiating insult as in a case of delayed graft function, particularly when patients are admitted for CT imaging and discharged within 24–48 h after the procedure. Third, we did not perform randomization, as actions for randomization for the type of CT with or without contrast were not feasible in this setting. Fourth, indication for CT (urgent vs. elective) reflects the clinical practice, and in some cases radiologists changed the mode taking into consideration the clinical scenario. In general, radiologists are relatively hesitant to perform CT with contrast in patients with impaired kidney function, and we do acknowledge that CT with contrast is performed on vital indication, as well as being fully aware of possible complications. However, we believe this is a strength of the study because it reflects daily clinical practice. Finally, the identification of periprocedural active infection, sepsis, or hemodynamic instability could not be retrieved directly from our database, and the precise volume and type of administered fluids could not be established. Furthermore, determination of eGFR on the basis of serum creatinine before and after imaging is limited in hospitalized patients with acute illnesses and with non-steady-state kidney function. Most importantly, there is likely a selection bias related to clinical judgment and decisions, for example, the avoidance of contrast-enhanced imaging in some patients with advanced renal functional impairment or performing contrast-enhanced imaging despite impaired renal function, considering the impact of enhanced imaging important enough to justify the administration of radiocontrast material, despite the risk of PC-AKI. In such patients, often with compound clinical conditions predisposing them to AKI, such as sepsis, hemodynamic instability, or exposure to other nephrotoxins, it may be impossible to differentiate the individual impact of a radiocontrast agent from the other clinical predisposing parameters, especially as PC-AKI is defined by the exclusion of other causes for deteriorating kidney function. The additional observation is that unwell patients with an urgent non contrast scan are at risk of AKI as a result of their clinical condition. The indications for CT are very different in the two groups, as are the baseline eGRF, and there were more urgent scans performed in the non-contrast group, with 43% having severe sepsis, which on its own may account for AKI. However, a prospective controlled study overcoming clinical judgment and decision-making on imaging strategies is likely not feasible; thus, our real-life data characterize clinical practice with a substantial component of uncertainties and possible confounders. Taking all these facts into consideration, we found that our real-world data provide an insight into the complicated problem of whether to perform CT with contrast or not, as well as trying to demystify the exaggerated threat of PC-AKI.

Moreover, due to the ambiguity of prophylaxis and current guidelines, in Poland, we cannot perform a contrast-enhanced computed tomography in patients with CKD on an outpatient basis. Unfortunately, therefore, patients may suffer from delayed diagnosis/treatment while waiting for hospitalization, and later to a hospital infection, often caused by multi-drug-resistant bacteria. In addition, we have repeatedly encountered a situation where non-enhanced CT was performed due to the fear of PC-AKI, but then contrast-enhanced CT was needed, exposing the patient to a higher radiation dose, because the previous examination was inconclusive. In general, patients with reduced glomerular filtration rate face the difficulties associated with performing CT with contrast. Internationally collected data points to the problem of not performing contrast-enhanced CT due to the fear of PC-AKI [13]. In the light of the new data, this fear seems to be ungrounded. The situation is similar in other risk groups in the field of oncology or transplantation, where caution needs to be exercised when administering iodinated contrast media, but it is not advisable to postpone the diagnosis because of fear of renal complications [31,32,33]. From our clinical perspective, if in CT with contrast will change the diagnosis, prognosis, and therefore outcome, it should be performed. Of course, and all necessary precautions should be taken. 

Contrast-induced nephropathy (CIN) is an important drawback following the administration of intravascular iodinated contrast agents. Hospitalized patients do have a large number of comorbidities and are treated with a variety of nephrotoxic medications (antibiotics, analgesics, chemotherapeutics, and others). Therefore, their risk for CI-AKI is higher than ambulatory patients. CT scan with contrast should be performed if indicated as its denial or avoidance may delay diagnosis and proper treatment. Moreover, CT scan without contrast is often inconclusive and needs subsequent CT with contrast. Preventive measures should be introduced to prevent or minimize the risk of CI-AKI. In the worst-case scenario, renal replacement therapy is a viable option.

### 3.2. Key Findings and Future Directions

AKI was more common in the non-enhanced CT population.

It appears that PC-AKI is not a great risk for patients, even those with chronic kidney disease.

The fear of using contrast agents seems to be exaggerated.

Prospective studies on large population with various stages of CKD are needed to prove or disprove the detrimental effects of contrast agents on kidney function.

## 4. Materials and Methods

In our study, we analyzed all computed tomography performed in our department in 2019. Patients were qualified for the study according to indication, taking into account clinical benefits, and not with regard to renal function. In each case, we recorded basic information characterizing the CT and the patient, referring to known potential factors increasing the risk of post-contrast acute kidney injury. We noted the type (with or without contrast), the scope of examination, and the mode in which the examination was performed (emergency or routine). As for patient-dependent factors, age, sex, and comorbidities were recorded, including the determination of kidney function by measurement of serum creatinine concentration and eGFR (calculated using CKD-EPI formula). Then, in each case, it was observed whether the parameters of renal function were checked after CT in three time periods: 1–7, 14–28, and over 28 days. It was not possible to trace previous parameters of kidney function in most cases as it was the first hospitalization or there was no adequate medical documentation. Cases where no follow-up examinations were recorded in any of the above-mentioned time periods were excluded from further analysis. Moreover, patients with end-stage renal disease treated with dialysis were excluded. 

The remaining cases were divided into two groups—patients undergoing contrast-enhanced CT and non-enhanced CT. In order to make the analysis more reliable, we reduced the larger group of patients so that the number of patients in each group was similar, and both groups had to represent similar criteria, such as age, sex, and comorbidities. After obtaining two clinically comparable groups, we recorded the number of cases with AKI in each instance. For this purpose, the KDIGO criteria were used (increase in serum creatinine concentration ≥0.3 mg/dL or ≥1.5–1.9 times the baseline value), in accordance with the applicable guidelines. Cases in which AKI developed later after CT than assumed in the definition of PC-AKI were also considered. The situations in which AKI developed later after CT than assumed in the definition of PC-AKI were also considered. This solution was chosen due to fact that in many cases did not undergo a follow-up examination 48–72 h after CT—our study is retrospective, and in Poland, parameters of renal function are not routinely monitored after CT, especially non-enhanced cases. It should also be taken into account that the increase in renal parameters occurs 48–72 after the administration of an iodinated contrast media; however, the duration of AKI is variable and may even cause permanent renal failure. Additionally, we concluded that if the contrast caused AKI significantly more often in our patients, we would notice it regardless of the assumed time frame. 

In each identified case of AKI after CT, a possible cause was searched for on the basis of the available medical documentation. As a result, more accurate information on the development of contrast-induced acute kidney injury in the study group was obtained.

## Figures and Tables

**Table 1:** Serum creatinine and eGFR in patients undergoing computed tomography with and without contrast.

	Before CT	1–7 Days after	14–28 Days after	>28 Days after
CT without contrast creatinine, mg/dL	1.16 (1.06; 10.61)	1.54 (1.12; 7.18) **##	1.44 (0.49; 12.98) *##	1.04 (0.21; 7.81)
CT without contrast eGFR, mL/min/1.73 m^2^	50 (5; 120) #	39 (7; 120) ##	45(4; 102) ##	72 (7; 120)
CT with contrast creatinine, mg/dL	0.99 (0.48; 8.73)	1.06 (0.33; 8.07)	0.96 (0.36; 7.85)	1.00 (0.49; 5.84)
CT with contrast eGFR, mL/min/1.73 m^2^	68 (7; 120)	66 (7; 120)	74 (7; 120)	70 (8; 120)

Data given are medians and minimum–maximum; * *p* < 0.05, ** *p* < 0.01 vs. baseline values before CT; # *p* < 0.05, ## *p* < 0.01 CT with vs. without contrast.

**Table 2:** Serum creatinine and eGFR in patients with AKI undergoing computed tomography with and without contrast.

	Before CT	1–7 Days after	14–28 Days after	>28 Days after
CT without contrast creatinine, mg/dL	1.22 (0.74; 10.61)	1.73 (0.70; 6.77) **	1.75 (1.00; 9.20) **	1.71 (0.93; 7.81) *
CT without contrast eGFR, mL/min/1.73m^2^	46 (9; 120)	31 (7; 86) **	34 (4; 69) **	31 (7; 116) *
CT with contrast creatinine, mg/dL	1.29 (0.67; 6.17)	1.46 (0.67; 6.53) **#	1.56 (0.64; 4.03) **#	1.77 (0.64; 4.74) *
CT with contrast eGFR, mL/min/1.73m^2^	53 (8;20) #	37 (7; 91) **#	40 (7; 87) #	34 (8; 116)

Data given are medians and minimum–maximum; * *p* < 0.05, ** *p* < 0.01 vs. baseline values before CT; # *p* < 0.05 CT with vs. without contrast.

**Table 3:** Comorbidities—number and percentage share (in parentheses).

Comorbid Disease	Participation in the Population Subjected to Contrast-Enhanced CT	Participation in the Population Subjected to Non-Enhanced CT
Cancer	72 (25.4%)	10 (14.9%)
Hypertension	181 (63.7%)	50 (74.6%)
Diabetes mellitus	77 (27.1%)	12 (17.9%)
COPD ^1^/asthma	39 (13.7%)	11 (16.4%)
Chronic heart failure	67 (23.6%)	24 (35.8%)
Coronary heart disease	58 (20.4%)	13 (19.4%)
Cirrhosis	18 (6.3%)	3 (4.5%)
Thyroid disease	6 (2.1%)	1 (1.5%)
Sepsis/severe infection	74 (26%)	29 (43.3%)
Anemia	169 (59.5%)	47 (70.1%)
Pulmonary embolism	21 (7.4%)	3 (4.5%)

^1^ COPD—chronic obstructive pulmonary disease.

**Table 4:** Indications for contrast-enhanced CT in the studied group of patients.

Indication	Number of Patients (*n* = 285)
Pulmonary embolism	21
Abscess	7
Neoplastic disease (diagnosis or stage assessment)	29
Vascular complications	3
Other	7

**Table 5:** Indications for non-enhanced CT in the studied group of patients.

Indication	Number of Patients (*n* = 67)
Vasculitis	6
Fracture/bone metastases	13
Stroke/intracranial bleeding	14
Sinusitis	6
Pneumonia/pulmonary fibrosis	16
Other	12

**Table 6:** The occurrence of AKI depending on the mode of CT.

Type of Examination		Urgent	Routine
Contrast-enhanced CT	Number of AKI cases	10	2
	Total number of CT	37	30
	AKI frequency	27%	6.7%
Non-enhanced CT	Number of AKI cases	12	5
	Total number of CT	43	24
	AKI frequency	27.9%	20.8%

**Table 7:** Relevant studies on the PC-AKI.

Study Group	Study Design	Study Procedures	Central Message	Additional Findings	Study Limitation	Other	Reference
NICIR study	Prospective	Serum creatinine within 48–72 h after the procedure	PC-AKI rate was 4.4% (95%CI: 1.4–9.9%) in the oral hydration arm and 5.3% (95%CI: 2.0–11.1%) in the i.v. hydration arm	Lower urine hepcidin at postoperative day 1 was an independent predictor for AKI development	Single-center Observational design Low rate of severe AKI and renal replacement therapy		[13]
KOMPAS trial	Prospective	CT with contrast in CKD stage 3	PC-AKI occurred in 11 patients (2.1%), including 7 of 262 (2.7%) in the no prehydration group and 4 of 261 (1.5%) in the prehydration group	The association between catalytic iron and adverse outcomes remained significant after adjusting for pRBC transfusions, suggesting that catalytic iron may be a more direct mediator of AKI than free hemoglobin	Observational design Modest sample size Enrollment of patients predominantly from surgical ICU Single-center study design No data on hepcidin and NGAL No urinary catalytic iron levels		[15]
Cleveland Clinic CKD registry	Registry	Serum creatinine within 48–72 h after the procedure	The incidence of AKI was 27% in the coronary angiography group, 24% in CT with contrast, and 24% in CT without contrast		Both coronary angiography and CT with contrast		[17]
17,934 visits to emergency department with CT (16,801 patients)	Single-center retrospective cohort study	Serum was collected before contrast exposure (baseline) and at 48–72 h following contrast exposure	AKI rate was similar between CT with and without contrast	AKI rate was not dependent on baseline kidney function; no difference with CKD rate, dialysis, and transplantation at sixth month AKI in 8.1% of non-CT group	Retrospective The volume of contrast used was not standardized and varied between patients		[18]
11,516 patients	Meta-analysis	Plasma samples were obtained on days 1 and 8, whereas hepcidin was measured on day 1 only	Higher plasma concentrations of catalytic iron and lower plasma concentrations of hepcidin were associated with a significantly greater risk of death	Increased transferrin saturation and higher concentrations of ferritin were also associated with death, but the magnitude of association was strongest for catalytic iron and hepcidin Lower transferrin was associated with higher catalytic iron concentrations	Observational nature of study Lack of data on cause of death Unknown markers of hemolysis Lack of data on intravenous iron administration, erythropoiesis-stimulating agents, and transfusion of packed red blood cells	Largest study to date assessing dysregulated iron homeostasis in the context of human AKI All patients enrolled in the study had AKI requiring RRT on enrollment Assessment of multiple iron parameters	[19]
4171 visits to ED, 1640 CT with contrast, 976 without contrast, and 1731 no CT at all	Single-center, propensity-matched, retrospective cohort study	Serum creatinine within 48–72 h after the procedure	The incidences of AKI were 7.2%, 9.4%, and 9.7% in those who underwent CECT, unenhanced CT, and no CT, respectively	Contrast administration was not associated with the increased risk of AKI	Only septic patients Heterogeneous group	Sepsis as a medical emergency was proven to benefit from early diagnosis and treatment initiation, often aided by CT with contrast	[19]
Enhanced MRI = 958, non-enhanced = 491, enhanced CT = 9576, non-enhanced CT = 11,660	Propensity score matching analysis	22,321 imaging studies	Patients with impaired kidney function have a greater risk of PC-AKI	Anemia and diabetes are risk factors for PC-AKI	Selection bias Retrospective data; creatinine taken 24–72 h after imaging	Creatinine takes up to 3 days before imaging	[21]
1009 patients form SCAPIS study	Prospective	Creatinine measurement in 2–4 after the angiography	Iohexol is safe in patients with eGFR > 50 mL/min	PC-AKI rate very low (0.2%); no effect of diabetes and NSAIDs use on AKI rate	Extension of blood sampling to 48-96 h while in ESUR criteria within 48–72 h Blood sampling before angiography 0–91 days (median 14 days)	Very homogenous and well-defined group aged 50–65 years	[22]
2583 CT scans in 2277 patients	Retrospective cohort analysis	The incidence of acute kidney injury (Acute Kidney Injury Network stages) and dialysis after acute kidney injury were assessed in the immediate period (24–48 h) and in a delayed period (72–96 h) after the scan.	AKI rate was not dependent on CKD stage	Dialysis after AKI was similar across eGFR subgroups.	Only 21 patients with CKD stage 5 and 47 with CKD stage 4 Restricted database; some comorbidities may be missing, as well as nephrotoxic drugs, contrast agent volume, and prophylaxis regimen		[34]
2008 on adult patients who underwent a contrast-enhanced computed tomography for urgent diagnostic purposes.	Single-center retrospective analysis	Creatinine assessment within 48 h	PC-AKI was a frequent complication (16.8%)	Sepsis, nephrotoxic drugs, and hemodynamic failure—risk factors for AKI PC-AKI associated with ICU mortality; need for renal replacement therapy in 29.2% of PC-AKI	Retrospective No data on contrast volume, no data on prophylaxis, Mixed medical-surgical ICU population Urgent procedure Repeated administrations of contrast were not assessed Fluid balance and hemodilution were not assessed		[35]
8 articles out of 2500 screened were analyzed	Systemic review (meta-analysis of observational studies)	Incidence of post-contrast acute kidney injury (AKI) following intravenous contrast agent administration	CT with contrast was not significantly associated with AKI.	Risk of contrast induced nephropathy (CIN) was negligible in patients with normal renal function, but the incidence appeared to rise to as high as 25% in patients with pre-existing renal impairment or in the presence of risk factors such as diabetes, advanced age, vascular disease, and use of certain concurrent medications	Systematic review addressed both CIN and PC-AKI because in literature the two terms CIN from PC-AKI were difficult to separate, even if these terms were not interchangeable	The incidence reported of AKI in patients undergoing cCT with contrast was not as high as thought before	[36]
67,831 patients older than 65 years of age (out of 186, 455 patients)	Meta-analysis (22 studies)	Incidence of AKI in elderly (over 65 years)	Incidence of CI-AKI was 13.6% in the elderly	The high incidence of CI-AKI in the elderly was consistent across different administration route subgroups (intracoronary contrast medium group, 15.5%; intravenous contrast medium group, 12.4%)	Incomplete data on risk factors for AKI Definitions of elderly and CI-AKI varied among the included studies, which brought heterogeneity No age-stratified subgroup analysis No KDIGO definition of AKI as vast majority of clinical trials on CI-AKI used the definition based on serum creatinine alone and without grading	No data regarding the impact of CI-AKI on a patient’s clinical course and prognosis, and no conclusive management strategy for the elderly are available	[37]
2240 cancer patients with eGFR < 45 mL/min undergoing CT with contrast (out of 6463 patients)	Observational retrospective	Creatinine measurement within 48–96 h after CT	AKI rate was 2.5%	eGFR, diabetes mellitus, and serum albumin level were risk factor for AKI	Retrospective Exclusion of 37% of eligible subjects (1298/3538) because creatinine levels immediately after CT were unavailable Diagnosis or prescription codes were used, but their accuracy in representing clinical information was not well validated	Development of the prediction model of AKI	[38]

## Data Availability

The data presented in this study are available on request from the corresponding author.
